# Use of Intravenous Immunoglobulins in Pediatric Viral Meningoencephalitis: A Real-World Retrospective Observational Study

**DOI:** 10.3390/jcm15114024

**Published:** 2026-05-22

**Authors:** Ilaria Lazzareschi, Mariachiara Mercuri, Ludovica Renzelli, Chiara Veredice, Marco Piastra, Anna Camporesi, Giulia Bersani, Cristina De Rose, Francesco Proli, Laura Martino, Andrea De Angelis, Rosa Morello, Barbara Fiori, Rosaria Santangelo, Roberta Onesimo, Danilo Buonsenso

**Affiliations:** 1 Area Pediatrica, Dipartimento di Scienze della Vita e Sanità Pubblica, Università Cattolica del Sacro Cuore, 00168 Roma, Italymartino.laura96@gmail.com (L.M.); roberta.onesimo@policlinicogemelli.it (R.O.); 2 Department of Woman and Child Health and Public Health, Fondazione Policlinico Universitario Agostino Gemelli IRCCS, 00168 Rome, Italy; 3 Medicine and Surgery, Università Cattolica del Sacro Cuore, 00168 Roma, Italy; ludovica.renzelli01@icatt.it; 4 Child Neurology and Psychiatry Unit, Department of Neuroscience, Università Cattolica del Sacro Cuore, Fondazione Policlinico Universitario Agostino Gemelli IRCCS, 00168 Rome, Italy; 5 Paediatric Intensive Care Unit and Trauma Centre, Policlinico Gemelli IRCCS, 00168 Rome, Italy; 6 Division of Pediatric Anesthesia and Intensive Care, ASST Grande Ospedale Metropolitano Milano, 20162 Milano, Italy; 7 Microbiology Unit, Fondazione Policlinico Universitario Agostino Gemelli IRCCS, 00168 Rome, Italy

**Keywords:** IVIG, encephalitis, viral, children

## Abstract

**Background:** Viral meningoencephalitis is a frequent cause of acute central nervous system infection in children, particularly in neonates and young infants. Although etiological diagnosis has improved through molecular testing, management remains largely supportive, and intravenous immunoglobulins (IVIGs) are occasionally used in clinical practice despite limited supporting evidence. **Methods:** We performed a single-center retrospective observational study including pediatric patients aged 0–10 years admitted between 2016 and 2025 with molecularly confirmed viral meningoencephalitis. Demographic, clinical, microbiological, therapeutic, and follow-up data were collected. Neurological outcomes and length of hospital stay were compared between patients treated with IVIG and those who were not. **Results:** Twenty-nine patients were included. Enterovirus was the most frequently identified pathogen (50.0%), followed by human herpesvirus (35.7%) and human parechovirus (14.3%). IVIG was administered to 28% of patients, all with enterovirus infection. IVIG-treated patients were significantly younger at presentation and more frequently presented with apnea (42.9% vs. 0%, *p* = 0.014). Most patients had a favorable neurological outcome (85.7%). Unfavorable outcomes, including neurodevelopmental delay and/or epilepsy, occurred in a minority of cases (14.3%) and exclusively in enterovirus-infected patients. No significant association was found between IVIG administration and neurological outcome. **Conclusions:** In this real-world pediatric cohort, IVIG use was associated with more severe clinical features rather than improved neurological outcomes, underscoring the need for careful consideration and further investigation in this setting, particularly in the subgroup of infants with enterovirus encephalitis.

## 1. Background

Viral meningoencephalitis is a major cause of acute central nervous system (CNS) infection in the pediatric population and represents a frequent reason for hospital admission, particularly among neonates and young infants [1,2]. In this age group, clinical presentation is often nonspecific and may include apnea, respiratory instability, feeding difficulties, seizures, and altered level of consciousness, making early assessment of disease severity and identification of patients at risk for long-term neurological sequelae particularly challenging [3,4,5].

Enteroviruses are the most common causative agents of viral CNS infections in neonates and young children and account for a wide range of clinical manifestations, from mild self-limiting disease to severe presentations associated with seizures, cardiorespiratory instability, and, in a minority of cases, persistent neurological impairment [6,7]. Human parechoviruses, particularly parechovirus type 3, have emerged as important pathogens in early infancy and are frequently associated with systemic and neurological symptoms despite minimal cerebrospinal fluid (CSF) pleocytosis [8,9]. Human herpesvirus 6 (HHV-6) is also commonly detected in CSF by molecular assays; however, its pathogenic role in CNS disease remains controversial, given the potential for viral latency or reactivation rather than primary neuroinvasive infection [10].

Advances in molecular diagnostic techniques, particularly the implementation of multiplex polymerase chain reaction (PCR) panels on CSF, have significantly improved the ability to identify viral pathogens in CNS infections [4,11]. Nevertheless, improved diagnostic yield has not been accompanied by a parallel expansion of therapeutic options. For most viral etiologies, management remains largely supportive, as specific antiviral therapies are available for only a limited number of pathogens [1,2]. As a result, clinical decision-making is often guided by disease severity and patient age rather than targeted etiological treatment.

In this context, intravenous immunoglobulins (IVIGs) are sometimes administered in clinical practice, particularly in younger patients or in those presenting with features perceived as more severe [12,13,14]. The rationale for IVIG use is based on their potential immunomodulatory and antiviral properties; however, evidence supporting a beneficial effect on neurological outcomes in pediatric viral meningoencephalitis is limited, heterogeneous, and largely derived from observational studies and case series [12,13,14]. Accordingly, current international guidelines do not recommend routine IVIG administration in viral encephalitis, reserving their use for selected cases or specific immune-mediated conditions [1,2,4].

Given the current therapeutic uncertainties, and variability in real-world clinical practice, we performed this study aimed at describing the patterns of IVIG use in pediatric viral meningoencephalitis and explore their association with short and middle-term neurological outcomes.

## 2. Objectives

The primary objective of this study was to evaluate the association between IVIG administration and the short and middle-term neurological outcomes at follow-up in children with viral meningoencephalitis.

Secondary objectives were:

1. To describe the epidemiological, clinical, laboratory and instrumental characteristics of a pediatric cohort with viral meningoencephalitis and cerebrospinal fluid positivity for enterovirus, human parechovirus or human herpesvirus 6.
2. To evaluate treatments and outcomes and identify clinical factors associated with IVIG administration.

## 3. Study Design and Methods

### 3.1. Study Design and Setting

A single-center retrospective observational study was conducted at Fondazione Policlinico Universitario A. Gemelli. Patients were identified through review of microbiological laboratory records and electronic medical charts. All pediatric patients aged 0–10 years admitted between January 2016 and December 2025 with a diagnosis of viral meningoencephalitis and cerebrospinal fluid (CSF) positivity for enterovirus (EV), human parechovirus (HPeV), or human herpesvirus 6 (HHV-6) were included.

### 3.2. Study Population and Case Definition

Pediatric patients admitted during the study period with clinical suspicion of central nervous system infection and cerebrospinal fluid (CSF) positivity for enterovirus (EV), human parechovirus (HPeV), or human herpesvirus 6 (HHV-6) detected by molecular assays were eligible for inclusion.

A case of viral meningoencephalitis was defined as the presence of clinical signs and symptoms consistent with central nervous system involvement (including fever, altered level of consciousness, seizures, apnea, or other neurological signs) in association with CSF positivity for one of the included viruses. Patients with evidence of bacterial central nervous system infection, documented by positive bacterial CSF cultures, were excluded. Additional exclusion criteria included lack of molecular confirmation on CSF and insufficient clinical documentation to allow a reliable case definition. Cases due to Herpers Simplex and Varicella–Zoster encephalitis were excluded, as a specific antiviral exists for these patients.

### 3.3. Data Collection

For each patient, the following data were systematically collected:

- Epidemiological data: age at onset, sex, season of admission.
- Medical history: prenatal and perinatal history and comorbidities.
- Clinical features at admission: fever, seizures, apnea, altered mental status, and other neurological or systemic signs.
- Laboratory findings: complete blood count, C-reactive protein (CRP), procalcitonin (PCT).
- Cerebrospinal fluid characteristics: chemical–physical examination of CSF.

### 3.4. Microbiological Investigations

Etiological diagnosis was established through molecular testing on CSF, including multiplex meningitis/encephalitis panels performed according to routine clinical practice. Results of blood, urine and CSF cultures, nasal swabs, and viral serologies on blood were also recorded when available.

### 3.5. Neurophysiological and Imaging Studies

During hospitalization, patients underwent instrumental investigations based on clinical indications, including:

- Electroencephalography (EEG);
- Cranial ultrasound;
- Brain magnetic resonance imaging (MRI).

Ophthalmological evaluation and hearing assessment (auditory brainstem responses [ABRs] and/or transient evoked otoacoustic emissions [TEOAEs]) were also recorded when performed.

### 3.6. Treatment

For each patient, administration of the following treatments was recorded:

- Intravenous immunoglobulins (IVIGs) (yes/no);
- Antibiotic therapy;
- Other supportive treatments.

The decision to administer IVIG was made by the treating clinical team based on individual clinical assessment and not according to a predefined treatment protocol.

### 3.7. Follow-Up and Outcome Definition

Neurological outcome was pragmatically classified as favorable or unfavorable based on available clinical documentation at follow-up visits. Outcome assessment was not based on standardized neurodevelopmental scales or formal neurological evaluations, but rather on routine clinical records. A favorable outcome was defined as a follow-up described as clinically normal or ongoing without reported neurological deficits.

An unfavorable outcome was defined as documented neurodevelopmental delay, epilepsy, or persistent neurological deficits. Follow-up duration was heterogeneous and not standardized.

### 3.8. Statistical Analysis

Continuous variables were described as mean ± standard deviation or median and interquartile range (IQR), according to data distribution. Categorical variables were reported as frequencies and percentages. Comparisons between IVIG-treated and non-treated patients were performed using the Mann–Whitney U test for continuous variables and Fisher’s exact test for categorical variables, given the small sample size. To identify factors associated with IVIG administration, given the small sample size and the risk of complete separation, Firth penalized logistic regression was used. A *p*-value < 0.05 was considered statistically significant. Analyses were performed on available cases, and no imputation for missing data was applied.

### 3.9. Ethics Committee

This is a subanalysis of a study on outcomes of neurological infections reviewed and approved by the Human Research Ethics Committee of the Fondazione Policlinico Universitario A. Gemelli IRCCS of Rome, Italy (ID 6307). The study was conducted in accordance with the Declaration of Helsinki and its subsequent amendments. No personal or identifiable data were collected during the conduct of this study. All parents of the children signed informed consent; they could choose not to participate in the study or to withdraw their consent at any time.

## 4. Results

### 4.1. Study Population and Baseline Characteristics

A total of 29 pediatric patients with molecularly confirmed viral meningoencephalitis due to enterovirus, human parechovirus, or HHV-6 were included in the study. Most patients were male (69.0%). The median gestational age at birth was 38.5 weeks (IQR 37.0–39.0), and the median age at symptom onset was 10 months (IQR 0.4–18 months). At presentation, fever was the most frequent symptom (80.8%), followed by seizures (30.8%) and apnea (11.5%).

Baseline laboratory parameters showed low inflammatory markers, with a median C-reactive protein level of 2.2 mg/dL (IQR 0.6–6.3) and procalcitonin levels within the normal range. The median white blood cell count at admission was 8.9 ×10^9^/L (IQR 6.5–11.3). Cerebrospinal fluid chemical–physical analysis showed median protein and glucose levels of 58.5 mg/dL (IQR 24.5–99.0) and 62.0 mg/dL (IQR 52.5–77.5), respectively. CSF pleocytosis was not consistently documented and was therefore not included in the analysis. Admissions occurred throughout the year, with a slight predominance during autumn (31.0%) and summer (27.6%). Most patients were previously healthy, and relevant pre-existing medical conditions were uncommon in the study population. Demographic, clinical, and laboratory parameters are summarized in Table 1.

### 4.2. Microbiological Findings

Molecular analysis of cerebrospinal fluid using multiplex PCR identified enterovirus as the most frequent pathogen, detected in 14 patients (50.0%), followed by human herpesvirus 6 (HHV-6) in 10 patients (35.7%) and human parechovirus in 4 patients (14.3%).

The detection of HHV-6 in cerebrospinal fluid should be interpreted with caution, as it does not necessarily indicate a causal role in central nervous system infection.

Bacterial cultures of cerebrospinal fluid were negative in all tested patients. Blood cultures were negative in the majority of cases (92.0%), with a small proportion not performed (8.0%). Urine cultures were negative in 13 patients (52.0%), not performed in 8 patients (32.0%), and positive in a minority of cases, yielding *Escherichia coli*, *Klebsiella* spp., *Enterococcus* spp., or polymicrobial growth. Stool cultures, when performed, were predominantly negative; enterovirus was detected in 4 patients (16.0%).

Respiratory viral testing was positive in 16 patients (66.7%) for enterovirus, while adenovirus, coronavirus, and *Mycoplasma pneumoniae* were each detected in single cases.

Microbiological findings of the study population are summarized in Table 2.

### 4.3. Diagnostic Investigations

Electroencephalography (EEG) was performed in 19 patients and showed normal findings in all evaluated cases. Cranial ultrasound was performed in 8 patients. Among them, 6 patients showed pathological findings, most commonly consisting of increased parenchymal echogenicity, while 2 patients had normal results. Brain magnetic resonance imaging (MRI) was performed in 23 patients. MRI findings were normal in 12 patients, whereas 11 patients presented pathological findings, predominantly represented by nonspecific signal alterations.

Ophthalmologic evaluation was performed in 8 patients and yielded normal results in all cases. Hearing screening with transient evoked otoacoustic emissions (TEOAE) and/or auditory brainstem response (ABR) testing was performed in 12 patients, with normal findings in all evaluated cases. Diagnostic investigations are summarized in Table 3.

### 4.4. IVIG Administration

Intravenous immunoglobulins (IVIGs) were administered to 7 patients (28.0%), while 18 patients (72.0%) did not receive IVIGs. All IVIG-treated patients had enterovirus infection. Compared with non-treated patients, IVIG-treated patients had a significantly lower age at symptom onset (10 days vs. 450 days, *p* = 0.001). Apnea was more frequently observed in the IVIG group (42.9% vs. 0%, *p* = 0.014), whereas seizures were not observed among IVIG-treated patients (0% vs. 44.4%, *p* = 0.046).

In univariable Firth-penalized logistic regression, apnea was significantly associated with IVIG administration (OR 28.8, 95% CI 1.3–628.9, *p* = 0.036) (Table 4). Other clinical and baseline variables were not significantly associated with IVIG use.

### 4.5. Neurological Outcomes and Follow-Up

**Figure 1:**
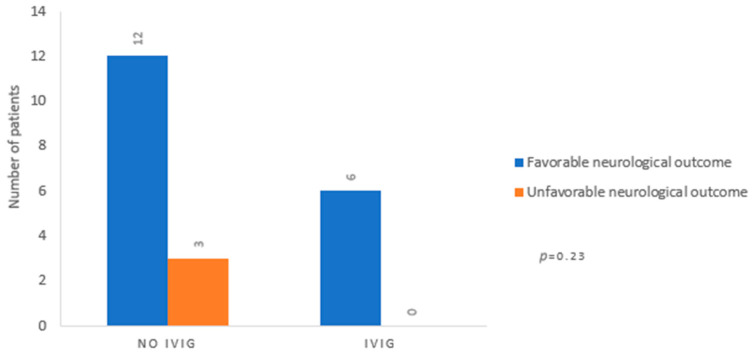
Neurological outcomes according to IVIG administration.

Median follow-up time was 1.21 years (IQR 0.68–3.19). Follow-up data were available for 21 patients (72.4%). Among these, 18 patients (85.7%) showed a favorable neurological outcome, while 3 patients (14.3%) developed pathological neurological outcomes during follow-up (Figure 1). Pathological outcomes included neurodevelopmental delay and/or epilepsy. Notably, none of the patients with pathological neurological outcomes had received IVIG during the acute phase of infection. Pathological neurological outcomes were observed exclusively in patients with enterovirus infection. No statistically significant association was found between IVIG administration and neurological outcome (*p* = 0.23).

### 4.6. Length of Hospital Stay

The median length of hospital stay for the overall study population was 9 days (IQR 7–11).

Patients treated with intravenous immunoglobulins (IVIGs) had a longer length of hospital stay compared with those who did not receive IVIG. However, no independent association between IVIG administration and length of hospital stay was demonstrated after adjustment for clinical and laboratory variables.

## 5. Discussion

In this retrospective observational study, we analyzed a pediatric cohort with viral meningoencephalitis, focusing on etiological agents, clinical features, and real-world use of intravenous immunoglobulins (IVIGs). Most patients experienced a favorable clinical course, regardless of IVIG administration, and no statistically significant association was observed between IVIG use and neurological outcomes.

Consistent with previous reports, enterovirus was the most frequently identified pathogen, followed by human herpesvirus 6 (HHV-6) and human parechovirus. All unfavorable neurological outcomes occurred in enterovirus-infected patients, confirming that, although often self-limiting, enteroviral central nervous system infections may be associated with long-term neurological sequelae, particularly in younger patients. In contrast, no adverse neurological outcomes were observed among patients with HHV-6 or parechovirus detection. However, the interpretation of HHV-6 positivity remains uncertain, as its detection in cerebrospinal fluid may reflect latency or reactivation rather than true neuroinvasive disease.

IVIG was administered to a minority of patients, predominantly younger infants and those presenting with apnea, likely reflecting differences in clinical presentation rather than disease severity. In neonates, nonspecific symptoms such as apnea often prompt a more cautious therapeutic approach. In our cohort, IVIG administration appeared to reflect clinical perception of risk rather than standardized treatment criteria. However, IVIG exposure was analyzed as a binary variable without detailed information on dosage, timing, or concomitant treatments. In addition, although IVIG is generally considered safe, rare neurological adverse events, including aseptic meningitis, have been reported and should be considered when evaluating IVIG use in clinical practice [15]. Baseline differences between treated and untreated patients may also have introduced confounding by indication. Therefore, the observed associations should be interpreted as exploratory rather than causal.

Our findings are consistent with previous studies that have not demonstrated a clear neurological benefit of IVIG in pediatric viral encephalitis outside immune-mediated conditions [14,16]. Although no unfavorable neurological outcomes were observed among IVIG-treated patients, this finding was not statistically significant and should be interpreted with caution given the small sample size and potential confounding factors [17].

The longer hospital stay observed in IVIG-treated patients likely reflects confounding by indication, as these patients were younger and clinically more complex.

This study has several limitations, including its retrospective single-center design, small sample size, and heterogeneous follow-up. Follow-up duration was not standardized and varied across patients. Moreover, the pragmatic definition of neurological outcome, while reflective of real-world clinical practice, may not capture subtle neurological deficits. In addition, the inclusion of only CSF PCR-positive cases may have introduced selection bias and limits the generalizability of our findings. Therefore, our conclusions should be interpreted within this diagnostically enriched cohort.

## 6. Conclusions

In this real-world pediatric cohort with viral meningoencephalitis, most patients experienced favorable neurological outcomes. IVIG administration was primarily associated with younger age and clinical presentation, particularly apnea, rather than with improved neurological outcomes. However, given the limitations of our study design and the lack of detailed treatment information, no firm conclusions can be drawn regarding the effectiveness of IVIGs. These findings should therefore be interpreted with caution and highlight the need for prospective multicenter studies to better define patient selection criteria and long-term outcomes associated with different management strategies.

## Figures and Tables

**Table 1:** Demographic and biochemical characteristics of 29 pediatric patients with viral meningoencephalitis.

Variable	Value
Sex, n (%)	
Male	20 (69.0)
Female	9 (31.0)
Gestational age at birth, weeks, median (IQR)	38.5 (37.0–39.0)
Age at symptom onset, months, median (IQR)	10 (0.4–18)
Symptoms	
Fever, n (%)	21 (80.8)
Seizures, n (%)	8 (30.8)
Apnea, n (%)	3 (11.5)
Laboratory parameters	
C-reactive protein at admission (mg/dL), median (IQR)	2.2 (0.6–6.3)
Procalcitonin at admission (ng/mL), median (IQR)	0.0 (0.0–0.6)
White blood cell count at admission, ×10^9^/L, median (IQR)	8.9 (6.5–11.3)
CSF examination performed, n (%)	29 (100)
CSF protein, mg/dL, median (IQR)	58.5 (24.5–99.0)
CSF glucose, mg/dL, median (IQR)	62.0 (52.5–77.5)
Season of admission, n (%)	
Spring	8 (27.6)
Summer	5 (17.2)
Fall	14 (48.3)
Winter	2 (6.9)

**Table 2:** Microbiological findings of the study population.

Microbiological Investigation	Result	N (%)
CSF multiplex PCR (FilmArray)		
Enterovirus	Positive	14 (50.0)
HHV-6	Positive	10 (35.7)
Human paraechovirus	Positive	4 (14.3)
CSF bacterial culture	Negative	25 (100)
Blood culture	Negative	23 (92.0)
	Not performed	2 (8.0)
Urine culture	Negative	13 (52.0)
	Positive	4 (16.0)
	Not performed	8 (32.0)
Stool examination	Negative	5 (20.0)
	Enterovirus detected	4 (16.0)
	Not performed	14 (56.0)
Respiratory viral testing	Positive - Enterovirus - Other viruses	19 (79.2) 16 (66.7) 3 (12.5)

**Table 3:** Diagnostic investigations of the study population.

Diagnostic Investigation	Performed, n	Normal Findings, n	Pathological Findings, n
Electroencephalography (EEG)	19	19	0
Cranial ultrasound	8	2	6
Brain MRI	23	12	11
Ophthalmologic evaluation	8	8	0
Hearing screening (TEOAE/ABR)	12	12	0

**Table 4:** Univariate logistic regression analysis of factors associated with IVIG administration.

Variable	N	β Coefficient	Odds Ratio (OR)	95% CI (OR)	*p*-Value
Gestational age (weeks)	21	−0.157	0.85	0.61–1.19	0.35
Comorbidities	25	0.577	1.78	0.31–10.2	0.517
Age at symptom onset (per day)	25	−0.007	0.99	0.99–1.00	0.077
Apnea	25	3.360	28.8	1.25–665	0.036
Seizures	25	−2.497	0.08	0.004–1.66	0.103
Lethargy	25	0.022	1.02	0.12–8.52	0.984
Irritability	25	0.99	2.69	0.24–30.7	0.426

Odds ratios and 95% confidence intervals were estimated using univariable penalized logistic regression with Firth’s correction to reduce small-sample bias and address potential separation. Sample size varied across analyses due to missing data. Results should be interpreted as exploratory.

## Data Availability

Dataset available upon request to the corresponding author.
